# Vitamin K Concentration and Cognitive Status in Elderly Patients on Anticoagulant Therapy: A Pilot Study

**DOI:** 10.1155/2020/9695324

**Published:** 2020-02-19

**Authors:** Ludovico Alisi, Clodomiro Cafolla, Alessandra Gentili, Sara Tartaglione, Roberta Curini, Arturo Cafolla

**Affiliations:** ^1^Ematologia, Dipartimento Biotecnologie Cellulari ed Ematologia, “Sapienza” Università, Roma, Italy; ^2^Physics Department, Durham University, Durham, UK; ^3^Dipartimento di Chimica, “Sapienza” Università, Roma, Italy; ^4^Dipartimento di Medicina Sperimentale, “Sapienza” Università, Roma, Italy

## Abstract

**Objectives:**

Recent studies have suggested that vitamin K may exert significant effects on the central nervous system. The present study investigates the relationship between vitamin K plasmatic levels and cognitive functions in elderly patients on oral anticoagulant therapy (OAT).

**Design:**

At the Thrombosis Centre of Haematology, “Sapienza” University of Rome, 85 patients on OAT, aged between 75 and 92, were randomly enrolled in the study. Patients were on OAT with vitamin K antagonists (VKAs). Vitamin K1 concentrations were determined using standardized High-Performance Liquid Chromatography (HPLC). Cognitive functions were assessed using the Milan Overall Dementia Assessment (MODA).

**Results:**

MODA scores are positively correlated to vitamin K1 concentration. Patients with vitamin K1 below 0.100 *μ*g/L and between 0.100 and 0.400 *μ*g/L and between 0.100 and 0.400 *μ*g/L and between 0.100 and 0.400 *p* < 0.001). Even long-term OAT (>10 years) does not affect MODA scores. Education seems to exert a greater role on the cognitive status in comparison with aging.

**Conclusions:**

The study shows a positive association between vitamin K1 concentration and cognitive status in elderly patients (≥75 years) on OAT. The relationship between vitamin K1 concentration and MODA scores is described by a linear model. Cognitive status is not influenced by the duration of OAT but by the years of education.

## 1. Introduction

Vitamin K includes a group of lipid-soluble molecules based on a common 2-methyl-1,4-naphtoquinone ring but with a different side chain at the 3-position [1–3]. Phylloquinone (2-methyl-3-phytyl-1,4-naphtoquinone), or vitamin K1, is mainly found in vegetables [1, 2]. A second class of vitamers is the menaquinones (MK). MKs are produced by bacteria as provitamins and present unsaturated 5-carbon (prenyl) side chains at the 3-position [1–3]. One of the menaquinones, MK-4, is not commonly produced by bacteria, but synthesised from vitamin K1 [2, 3]. Vitamin K has a key role in the carboxylation of glutamate residues in proteins leading to gamma-carboxyglutamate (Gla) residues. Gla residues bind calcium and are essential for the activity of the so-called Gla proteins. Gla-proteins are involved in blood coagulation (coagulation factors II, VII, IX, and X and protein C, S, and Z), as well as in bone and vascular metabolism (osteocalcin and growth arrest-specific protein 6, Gas-6, respectively) [1, 2].

Vitamin K has been found in rat and human brain. Here, the most common vitamer is MK-4 [3, 4]. These findings have contributed to an increasing interest in the role of vitamin K in cognitive impairment and neurodegenerative diseases. Recent studies seem to suggest that vitamin K may exert some significant effects on the central nervous system (CNS) [3]. Different studies, however, show discrepancies on whether vitamin K would act as a protection factor against cognitive impairment or would contribute to neurodegeneration and loss of cognitive functions. On the one hand, Gas-6 helps the development and survival of many central nervous system's cellular lines [5]. On the other hand, Gas-6 has been shown to inhibit, in a concentration-dependent manner, the overexpression of Tyro3. Tyro-3 is a tyrosine kinase which reduces *β*-amyloid production [6].

Vitamin K may influence cognitive functions through other vitamin K-dependent proteins. Protein S, for example, seems to act as a protective factor during ischemic damage [7] and as a preserving factor for the integrity of the blood-brain barrier [8].

Vitamin K seems to regulate multiple enzymes involved in the sphingolipid biosynthesis [3, 9, 10]. Sphingolipids are present in high concentrations in brain tissues where they are important membrane constituents and major signalling molecules [10]. Their altered metabolism is involved in the pathological mechanisms leading to *β*-amyloid accumulation [11] as well as in the development of cognitive impairment in murine models [12, 13].

In the last five years, clinical observational studies have started investigating the impact of vitamin K on cognitive functions [14–16]. An increase in the dietary vitamin K intake has been shown to improve cognitive performance in geriatric patients [14, 15]. This is confirmed by the fact that a decrease in serum concentrations of vitamin K is associated with deterioration in verbal episodic memory [16]. A case control study conducted by our group on the relationship between vitamin K and the percentage of time in therapeutic range (TTR%) confirmed that subjects with low vitamin K1 plasmatic concentrations (below 0.060 *μ*g/L) were more likely to show signs of neurodegenerative diseases [17].

The impact of vitamin K on cognitive functions is indirectly confirmed by the effects of vitamin K antagonists (VKAs) which interfering with vitamin K metabolism seem to worsen neurodegenerative diseases. Rats treated with VKAs have shown altered MK-4 and sphingolipids levels in the brain, as well as cognitive and behavioural disorders [13]. Notably, patients on oral anticoagulant therapy (OAT) with VKAs have shown brain volume abnormalities [18].

Although only a small number of evidences are currently available, they seem to suggest that vitamin K may play a crucial role in the biochemistry and pathophysiology of the CNS. One key point to be explored in human subjects is whether vitamin K levels are related to the overall cognitive abilities and mental impairment, rather than just to some aspects of the cognitive performance. Given the increasing number of elderly subjects on OAT [17], it is also crucial to confirm any impacts anticoagulant drugs may have on vitamin K levels and cognitive functions. This may shine a light on the multifactorial pathogenesis of currently incurable neurodegenerative diseases. The present study, hence, investigates the relationship between plasmatic concentrations of vitamin K1 and the overall cognitive functions in elderly patients on OAT.

## 2. Materials and Methods

### 2.1. Patients

At the Thrombosis Centre of Haematology, “Sapienza” University of Rome, 625 OAT patients older than 75 years are regularly followed as outpatients. Of these, 456 patients are on OAT with VKAs or with direct oral anticoagulants (DOACs).

From 1 November 2016 to 15 December 2017, a random sample of patients has been selected for the study. Of 456 possible patients, 105 were chosen and gave their informed consent to participate in the study. The selection method was that the first patient who underwent a prothrombin time/international normalized ratio (PT/INR) test or other coagulative tests for each hour between 8 a.m. and 11 a.m., from Monday to Wednesday, was enrolled. Patients were excluded if their alcohol assumption was higher than a single serving/day, or if they showed evidences of an active hepatopathy. We excluded 12 subjects who were on OAT with DOACs. This is due to the fact that the limited number of patients treated with DOACs would not allow any statistically significant analysis. The final cohort was made up of 85 patients.

Education background significantly varied ranging from less than 4 years of studies (lack of primary school diploma) to more than 17 years (equivalent to university degree).

These 85 patients (52 males and 33 females) were aged between 75 and 92 (mean = 83.4 ± 0.4) and were on OAT with VKAs (either warfarin or acenocoumarin).  Table 1 shows the distribution of patients by therapeutic indication. OAT quality was assessed by TTR%. TTR% was calculated according to the Rosendaal method [19], considering PT/INR values over the last 9 months. The PT/INR target was 2.5 (range: 2.0–3.0) in patients with atrial fibrillation, deep venous thrombosis, pulmonary embolism, and arterial disease [17]. The PT/INR target was 3.0 (range: 2.5–3.5) in those with mechanical heart prosthesis [17].

### 2.2. Specimen Collection and Vitamin K1 Dosage

Blood collection was performed after an overnight fast. Peripheral blood samples were obtained by venous puncture. Samples were collected in three vacutainer tubes containing 0.129 M sodium citrate as an anticoagulant and centrifuged at 2700 g for 10 minutes. Plasma was extracted, aliquoted in Eppendorf tubes, and stored at −80°C until further analysis.

Vitamin K1 analysis was conducted in the Department of Chemistry, Sapienza University of Rome, using standardized High-Performance Liquid Chromatography (HPLC) procedures. HPLC was performed with a micro HPLC/autosampler/vacuum degasser system PE Series 200 (Perkin Elmer, Norwalk, CT). Analytes were detected and quantified by a 4000 Qtrap® (AB SCIEX, Foster City, CA, USA) mass spectrometer, using liquid chromatography-hybrid quadrupole linear ion trap mass spectrometry. The lowest limit of quantification (LLOQ) for vitamin K1 is 0.060 *μ*g/L. The concentration values are affected by an error equal to 13% of the values themselves. The method may, however, show a lower precision for values below 0.100 *μ*g/L [20]. The method had already been validated according to the Food and Drug Administration (FDA) guidelines with its accuracy evaluated through participation in the Vitamin K External Quality Assessment Scheme (KEQAS) [20].

### 2.3. Cognitive Assessment

Different tests are currently available to investigate cognitive functions and abilities. The most widely used test is the Mini Mental State Examination (MMSE) [21]. This is a rapid 30-point questionnaire which does not require any training and provides reliable results in the diagnosis of cognitive impairment. MMSE, however, has a significantly low sensitivity in identifying Mild Cognitive Impairment (MCI) [22]. Therefore, we decided to use the Milan Overall Dementia Assessment (MODA) [22–25]. MODA is a test with a significantly higher sensitivity in the detection of signs of MCI [22]. Cognitive evaluation of the patients was performed after the venous puncture.

The MODA test is based on a three stages examination:

1. *Autonomy Scale*. This is found altered only in the most deteriorated patients
2. *Orientation Enquiry*. This part of the test investigates 4 different orientation areas: temporal, spatial and personal orientation, and family relationships
3. *Neuropsychological Tests*. This investigation evaluates the levels of attention, memory, intelligence, space cognition, visual perception, and language

We used age and years of school correction tables to obtain the final score for each patient. According to the protocol's instructions, cognition values were considered normal if the score was ≥85/100 and pathological below 80/100. Values between 80 and 85 are considered borderline. Even if some authors quote MODA score up to one decimal place [22, 24, 25], we took the error on the MODA to be ±1. This was performed so as to avoid any potential overestimation in the precision of the method.

### 2.4. Data Analysis

Data was retrieved and statistically analysed using a specific program, which was developed by one of the authors using Python programming language. The program evaluated the correlation between MODA scores and vitamin K1. Data were analysed considering a threshold value for the vitamin K1 of 0.060 *μ*g/L. This value is the LLOQ for the HPLC method [20]. Advanced analysis was conducted binning the data according to the vitamin K centiles (0.100–0.199 *μ*g/L, 0.200–0.299 *μ*g/L, and so on) and bicentiles (0.100–0.299 *μ*g/L, 0.300–0.499 *μ*g/L, and so on). For each bin, the MODA score and the corresponding error were taken to be the mean and the standard error, respectively. When binning the data, vitamin K concentrations below 0.100 *μ*g/L were excluded. This was performed so as to remove any potential bias among centiles. The centile from 0 to 0.100 *μ*g/L would be significantly different from the others. The lower detection limit for vitamin K1 being 0.060 *μ*g/L, this centile would not include any values coming from the first half of the centile itself. Furthermore, the HPLC method is potentially affected by lower precision for concentrations below 0.100 *μ*g/L [20]. A further analysis was conducted according to whether the patients had vitamin K1 plasmatic levels below or above 0.400 *μ*g/L.

Data analysis was performed using Pearson's chi-squared, *χ*^2^, test. The *χ*^2^ test was implemented into our custom-made Python program. The relationship between MODA scores and vitamin K1 levels was evaluated considering the reduced *χ*^2^ (*χ*^2^_*ν*_). For a system with *ν* degrees of freedom, the goodness of the fit is proved for *χ*^2^_*ν*_ ≈ 1. The other condition to test is whether *χ*^2^ is within ±2 standard deviations of the mean, that is, within the range $\nu \pm \;2\sqrt{2\nu }$. The null hypothesis of a relationship between the two quantities is further tested by the cumulative probability function (P). The significance threshold being set to less than 0.001 (*p* < 0.001), P needs to be greater than *p* so as to not reject the null hypothesis [26, 27]. Further data analysis on the goodness of the linear fits is performed assessing the regression with the Pearson correlation coefficient (*r*). A value for *r* of 1 shows that the linear regression predictions perfectly match the observed data [27, 28]. We evaluated also the correlations between, on the one hand, MODA scores and vitamin K1 levels, and, on the other hand, education, age, OAT length, and comorbidities. Also here, we set a threshold value for the vitamin K1 of 0.060 *μ*g/L, corresponding to the LLOQ for the HPLC method [20].

## 3. Results

The characteristics of all the patients are reported in Table 1. Out of the 85 patients on OAT, 52 were males and 33 females. Out of the entire cohort, 71 (84%) patients were aged 80 years or above. Atrial fibrillation was the most common diagnosis among patients (49%) followed by mechanical heart prosthesis (34%) and by deep venous thrombosis or pulmonary embolism (8%). Patients enrolled in the present study had been receiving OAT for at least 18 months and 65 (76%) patients for more than 10 years.

As shown in Table 2, vitamin K1 concentration was below 0.100 *μ*g/L in 27 patients and equal to or above 0.100 *μ*g/L in 58 patients. Considering the patients with vitamin K1 concentration above 0.100 *μ*g/L, 32 of them had vitamin levels between 0.100 and 0.400 *μ*g/L; the remaining 26 had vitamin concentrations above 0.400 *μ*g/L.

TTR% tends to increase with vitamin K concentration (Table 2). Patients with vitamin K levels below 0.100 *μ*g/L had a mean TTR% of 57 (±6) %. For vitamin K levels between 0.100 and 0.400 *μ*g/L and above 0.400 *μ*g/L, the mean TTR% was 66 (±4) % and 61 (±3) %, respectively. These results are consistent with previous observations [17].

Patients' MODA scores are reported in Table 3. The percentage of MODA scores above 90 was higher in patients younger than 85 years in comparison with older subjects (38% vs. 29%, respectively). The frequency of MODA scores below 80 was almost the same in subjects younger and older than 85 (22% vs. 20%, respectively).

The higher MODA scores in subjects younger than 85 years would suggest an impact of aging on the deterioration of cognitive functions. No clear correlation was however found between cognitive functions and age ([Supplementary-material supplementary-material-1] in the Supplementary Material); this is not surprising given the age correction factors applied to the MODA scores. Also the OAT length does not show any clear correlation to the vitamin K levels and cognitive functions (see Supplementary Material Figures [Supplementary-material supplementary-material-1] and [Supplementary-material supplementary-material-1], respectively). Education instead seems to exert a greater influence on the cognitive status. The MODA score was below 80 in 3 (9%) subjects with at least 13 years of school and in 11 (41%) patients with less than 7 years of school. This is further supported by [Supplementary-material supplementary-material-1] showing that, for increasing years of school, the dispersion of MODA scores tends to decrease and small values are less frequent.

**Figure 1:**
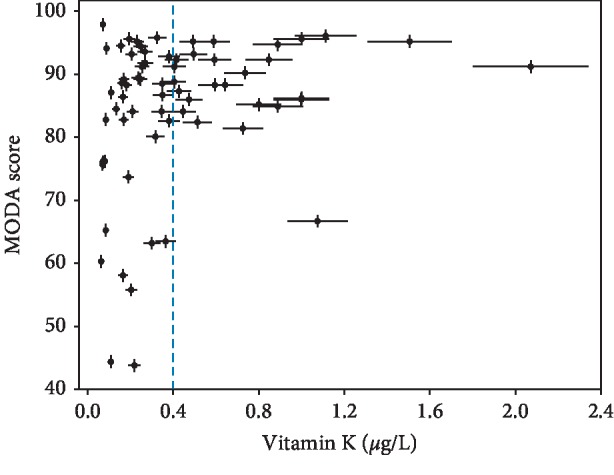
MODA scores vs. vitamin K1 plasmatic levels. The dispersion of the values tends to decrease as the vitamin K1 concentration increases (see also [Supplementary-material supplementary-material-1] in the Supplementary Material). This leads to larger mean MODA scores for higher concentrations of the vitamer, in particular for concentrations above 0.400 *μ*g/L (blue dashed line).

**Figure 2:**
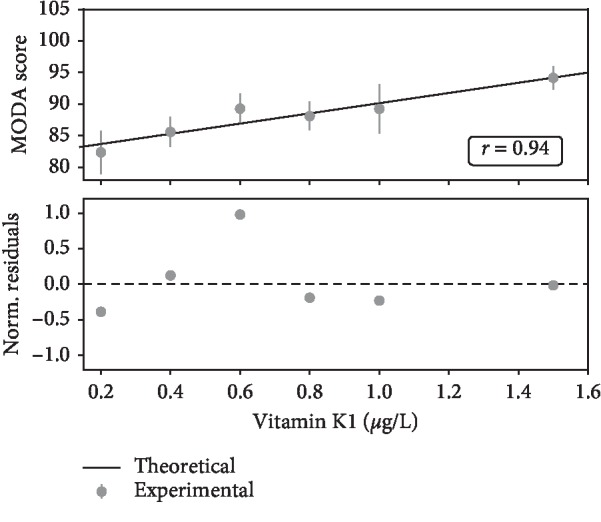
Analysis of MODA scores vs. vitamin K1 levels after binning the data. The linear relationship between MODA scores and vitamin K1 plasmatic levels appears clear after binning the data according to vitamin K1 concentration bicentiles. The value of *χ*^2^_*ν*_ of 0.3 is not <<1 thus suggesting that we should not reject the null hypothesis of a linear relationship between vitamin K1 levels and MODA scores. This is supported by the normalised residuals being randomly distributed around the zero and not showing any clear structures. The value for the Pearson correlation coefficient of 0.94 shows a very high positive correlation between MODA scores and vitamin K levels, thus further confirming that the linear regression is a good model for the data.

As shown in Table 2, MODA scores are correlated to vitamin K1. Patients with vitamin K1 below 0.100 *μ*g/L had a mean MODA value of 79 ± 5. For vitamin K1 levels between 0.100 and 0.400 *μ*g/L, patients showed a slight increase in the mean MODA score (82 ± 3). Patients with vitamin K1 above 0.400 *μ*g/L had a significantly greater mean MODA value (89 ± 1). Cognitive functions seem to be related to vitamin K1 levels by a logarithmic-like function with MODA scores dispersion decreasing for increasing levels of vitamin K1 (Figure 1). This is confirmed by further analysis dividing the patients according to vitamin K1 concentration centiles ([Supplementary-material supplementary-material-1] in the Supplementary Material). For the low concentration centiles, MODA scores show a large variability, that is, standard deviation (SD) > 10. An increase in vitamin K1 above 0.400 *μ*g/L corresponds to a decrease in SD below 10 (see also Figure 1). Therefore, 0.400 *μ*g/L was taken to be our threshold value. Given that some centiles have a very small number of patients, further analysis was performed binning the data into bicentiles. This is pictorially represented in Figure 2 showing a linear relationship between vitamin K1 concentrations and cognitive capacities. The goodness of the fit is confirmed by *χ*^2^ and *χ*^2^_*ν*_ being 1.21 and 0.3, respectively. Here, *χ*^2^ is within 2 standard deviations of the mean, and *χ*^2^_*ν*_ is close to the ideal value of 1. The hypothesis of a linear relationship between vitamin K1 levels and cognitive functions is statistically significant (*p* < 0.001) as shown by the calculated value for the cumulative probability function *P* =0.85 being close to the ideal value of 0.5 [26, 27]. The *r* value of 0.94 shows a high positive correlation between vitamin K levels and MODA scores. This further confirms that the linear regression is a valid model to fit the data [27, 28].

The results are particularly remarkable considering that there do not seem to be any clear relations between, on the one hand, comorbidities and, on the other hand, vitamin K plasmatic concentration and cognitive functions (see Tables [Supplementary-material supplementary-material-1] and [Supplementary-material supplementary-material-1] and in Figures [Supplementary-material supplementary-material-1]–[Supplementary-material supplementary-material-1] in the Supplementary Material).

## 4. Discussion

The role of vitamin K in coagulation processes has been extensively studied and characterised [29]. Recent studies have suggested that the vitamin may also play a key role in cognitive performance [13–16]. Studies on animal models have suggested that vitamin K may be involved in memory consolidation. Rats fed with a low vitamin K diet presented an altered sphingolipid profile in their hippocampus, which is the key cerebral region for memory [13]. Clinical studies on human subjects confirmed a positive association between, on the one hand, serum concentrations and dietary intake of vitamin K and, on the other hand, cognitive outcomes in healthy elderly [14–16]. The large number of geriatric patients on OAT has suggested the need for further investigations so as to understand whether treatments with any vitamin K antagonists may affect cognitive functions.

The present study is the first one, to the best of our knowledge, that analyses the relationship between vitamin K1 levels and cognitive performance in elderly patients on OAT with VKAs. Previous studies mainly focused on animal models or human tissues analysed post mortem [30]. Some investigations had evaluated the relationship between vitamin K and cognitive functions in human subjects [30]. These studies, however, relied on simple cognitive tests, such as the MMSE. Here, the cognitive functions of the patients have been assessed using a more accurate test, that is, the MODA score. The MODA is a complex tool which allows evaluating the levels of attention, memory, intelligence, space cognition, visual perception, and language. The MODA has shown significantly high sensitivity in the detection of MCI signs [22].

This study shows a positive association between plasmatic levels of vitamin K1 and cognitive status in patients older than 75 years on OAT. There is a linear relationship between vitamin K1 concentration per bicentile and cognitive functions as measured by the MODA score. The MODA values tend to be borderline or pathological for vitamin K1 concentrations smaller than 0.400 *μ*g/L. This would support the role of vitamin K in ensuring and preserving cognitive functions. Vitamin K1 concentration of 0.400 *μ*g/L may be a threshold value below which important signs of cognitive impairment appear. The reasons behind a positive impact of vitamin K on cognitive status may be explained with the fundamental role of this vitamin on the synthesis of protein S [7, 8] and sphingolipids [3, 9, 10]. These molecules have been shown to have a key role in ensuring brain signalling and metabolism [7–10, 30].

Interestingly, the cognitive capacities do not correlate with the anticoagulant therapy and with its duration. These findings would suggest that long-term OAT can be prescribed to elderly patients without any major impacts on their cognitive status.

Education seems to have a greater influence on cognitive functions in comparison with aging. Despite applying correction factors for both education and aging, the former still seems to influence MODA scores. Only 9% of subjects with high education had a MODA score below 80, whereas low education levels are associated to a pathological MODA value in 41% of the patients.

Further studies on larger cohorts of patients are needed so as to confirm the findings of this investigation.

## 5. Conclusions

This pilot study has shown a positive correlation between vitamin K1 concentration and cognitive status. This is in particular true for vitamin K1 concentrations above 0.400 *μ*g/L. A multicentre research is strongly recommended so as to confirm the data of the present investigation.

## Figures and Tables

**Table 1:** Characteristics of patients according to the diagnosis.

Diagnosis		AF	MHP	DVT/PE	AD	Total
Patients (number)		42	29	7	7	85

Sex	Male	26	18	5	3	52
	Female	16	11	2	4	33

Age (years)	75–80	4	6	3	1	14
	80–84	15	16	4	3	38
	85–89	20	6	0	3	29
	>89	3	1	0	0	4

Time of therapy (months)	<24	3	0	0	0	3
	24–60	3	0	1	0	4
	61–120	6	2	4	1	13
	>120	28	27	5	5	65

AF: atrial fibrillation. MHP: mechanical heart prosthesis. DVT: deep venous thrombosis. PE: pulmonary embolism. AD: arterial disease.

**Table 2:** MODA scores and percentage of time in therapeutic range (TTR%) according to the plasmatic levels of vitamin K1. For both the MODA score and the TTR%, the value and the associated error are the mean and the standard error, respectively.

Vitamin K1 (*μ*g/L)	<0.060	<0.100	0.100–0.400	>0.400	Total
TTR (%)	59 ± 4	57 ± 6	66 ± 4	61 ± 3	—
MODA score	81 ± 4	79 ± 5	82 ± 3	89 ± 1	—
Pts (number)	19	8	32	26	85

**Table 3:** MODA scores according to patients' characteristics.

MODA score		>90	90–85	84–80	79–70	<70	Total
Sex	Male	24	15	7	4	2	52
	Female	6	9	7	3	8	33

Age (years)	75–80	4	5	1	2	2	14
	80–84	15	9	5	2	5	36
	85–89	10	8	7	3	3	31
	>90	0	2	1	0	1	4

Education (years of school)	<4	1	1	2	0	3	7
	4–7	5	5	2	2	6	20
	8–12	11	6	5	3	1	26
	13–16	7	6	4	1	1	19
	>16	5	6	1	1	0	13

## Data Availability

The data used to support the findings of this study are available from the corresponding author upon reasonable request.

## Supplementary Materials

Supplementary Materials Supplementary materials include Figure S1 (MODA score vs. age of the patients), Figure S2 (vitamin K levels vs. therapy length), Figure S3 (MODA score vs. therapy length), Figure S4 (MODA score vs. education), Table S1 (mean values and standard deviations of MODA scores for vitamin K1 centiles), and Table S2-3 and Figures S5-8 (MODA scores and vitamin K levels vs. comorbidity).
