# Supplementary material for: Vitamin K Concentration and Cognitive Status in Elderly Patients on Anticoagulant Therapy: A Pilot Study
Source: J Aging Res. 2020 Feb 19;2020:9695324. doi: 10.1155/2020/9695324 (PMC7049843; doi:10.1155/2020/9695324)
Supplement: Supplementary Materials — include Figure S1 (MODA score vs. age of the patients), Figure S2 (vitamin K levels vs. therapy length), Figure S3 (MODA score vs. therapy length), Figure S4 (MODA score vs. education), Table S1 (mean values and standard deviations of MODA scores for vitamin K1 centiles), and Table S2-3 and Figures S5-8 (MODA scores and vitamin K levels vs. comorbidity). [file 9695324.f1.pdf]

Supplementary Material

Vitamin K concentration and cognitive status in elderly patients on anticoagulant therapy: a pilot study

Ludovico Alisi,[1*] Clodomiro Cafolla,[2*] Alessandra Gentili,[3] Sara Tartaglione,[4] Roberta Curini,[3] and Arturo Cafolla[1]

[1] Ematologia, Dipartimento Biotecnologie Cellulari ed Ematologia, "Sapienza" Università, Roma, Italy.
[2] Physics Department, Durham University, Durham, UK.
[3] Dipartimento di Chimica, "Sapienza" Università, Roma, Italy.
[4] Dipartimento di Medicina Sperimentale "Sapienza" Università, Roma, Italy.

*These authors contributed equally to this work.

Correspondence should be addressed to Arturo Cafolla; arturo.cafolla@uniroma1.it

Index of content:

1. MODA scores and age
2. Vitamin K levels and MODA scores vs oral anticoagulant therapy length
3. MODA scores and years of education
4. MODA scores: mean, standard deviation and standard error per vitamin K centile
5. MODA scores and vitamin K levels vs comorbidities
6. Supplementary references

1. MODA scores and age

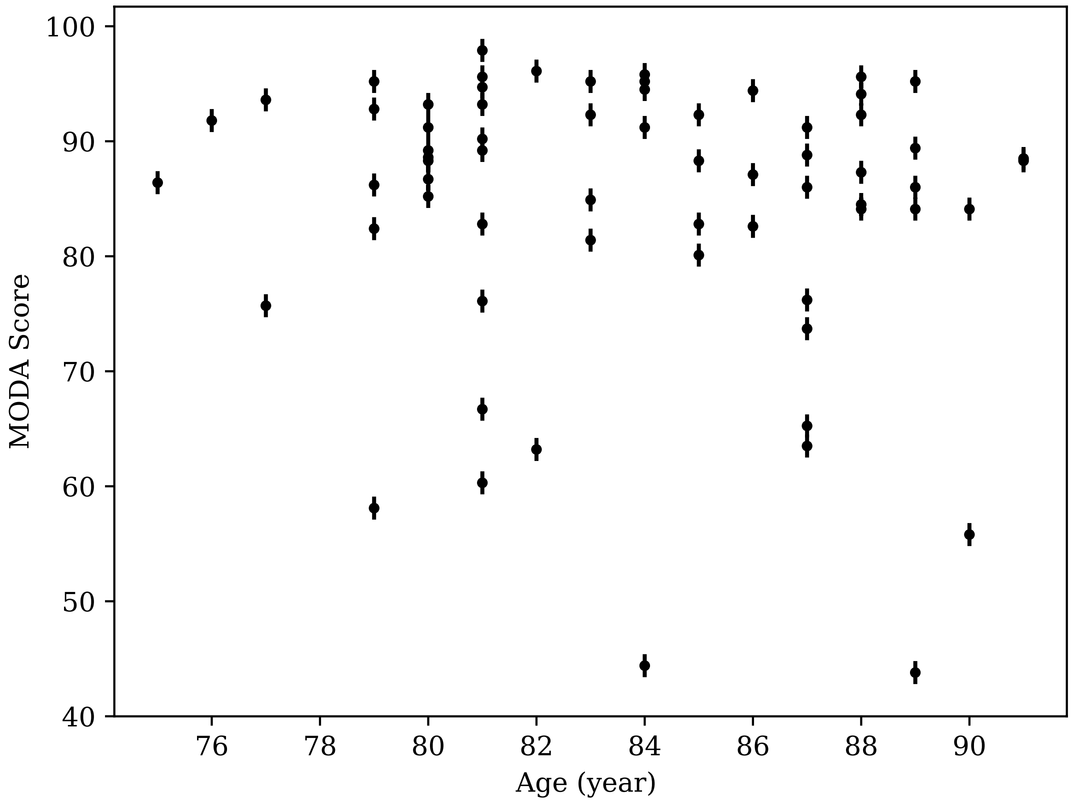

Figure S1. No clear relationship can be identified between MODA scores and age of the patients. MODA scores indeed show a random scatter. This finding is expected as age correction factors were applied to the MODA scores.

Fig. S1 shows that there does not seem to be any clear trend between MODA scores and aging. Given the small sample size, further studies on larger cohorts and comparing patients on OAT vs healthy subjects are needed in order to confirm the present findings.

2. Vitamin K levels and MODA scores vs oral anticoagulant therapy length

As shown in Figs S2 and S3, the duration of oral anticoagulant therapy (OAT) does not show any clear impact on vitamin K levels and MODA scores. Both vitamin K concentrations and MODA scores are randomly scattered. There is however a tendency for MODA scores to have larger values and to be less dispersed as the OAT length increases (Fig. S3). The data therefore seems to suggest, at least, that the duration of OAT with vitamin K antagonists (VKAs) would not exert any significantly negative effects on cognitive functions, as measured by MODA scores. This finding is particularly interesting considering that some studies on animal models and human subjects suggested a negative impact of VKAs on cognitive functions [1-2]. The data is however limited and further studies are needed to confirm such a trend.

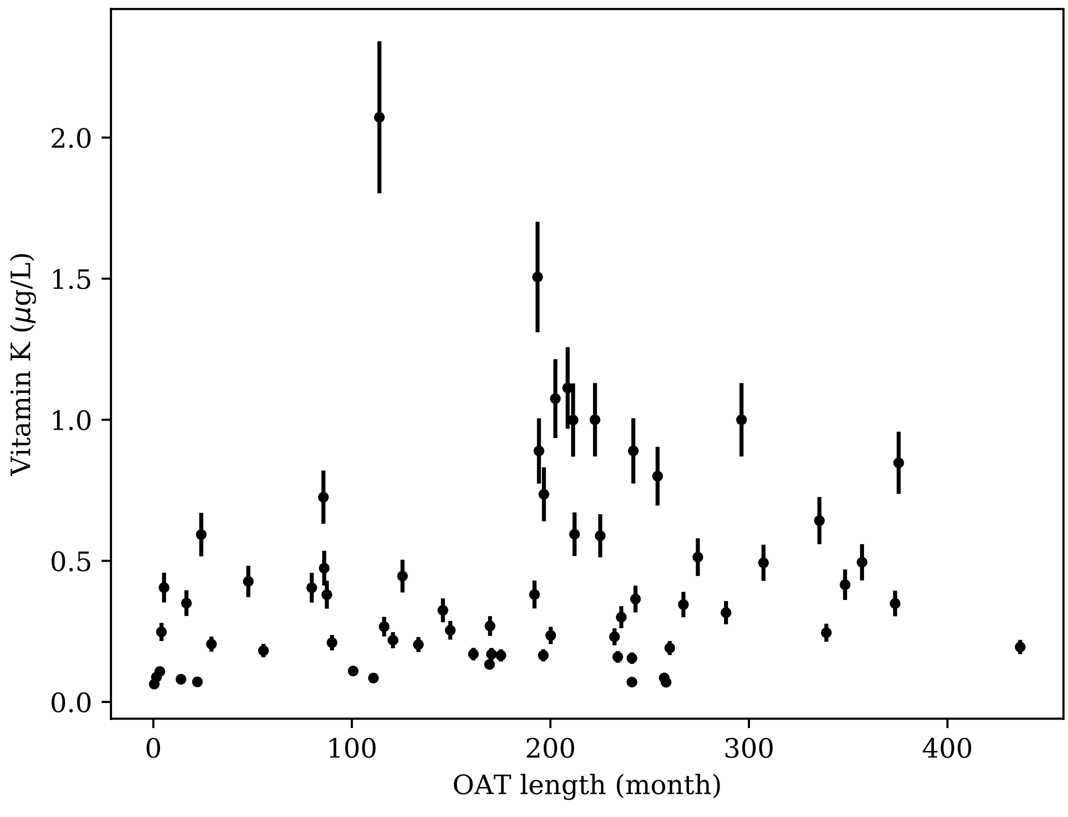

Figure S2. No clear relation can be identified between vitamin K levels and OAT length.

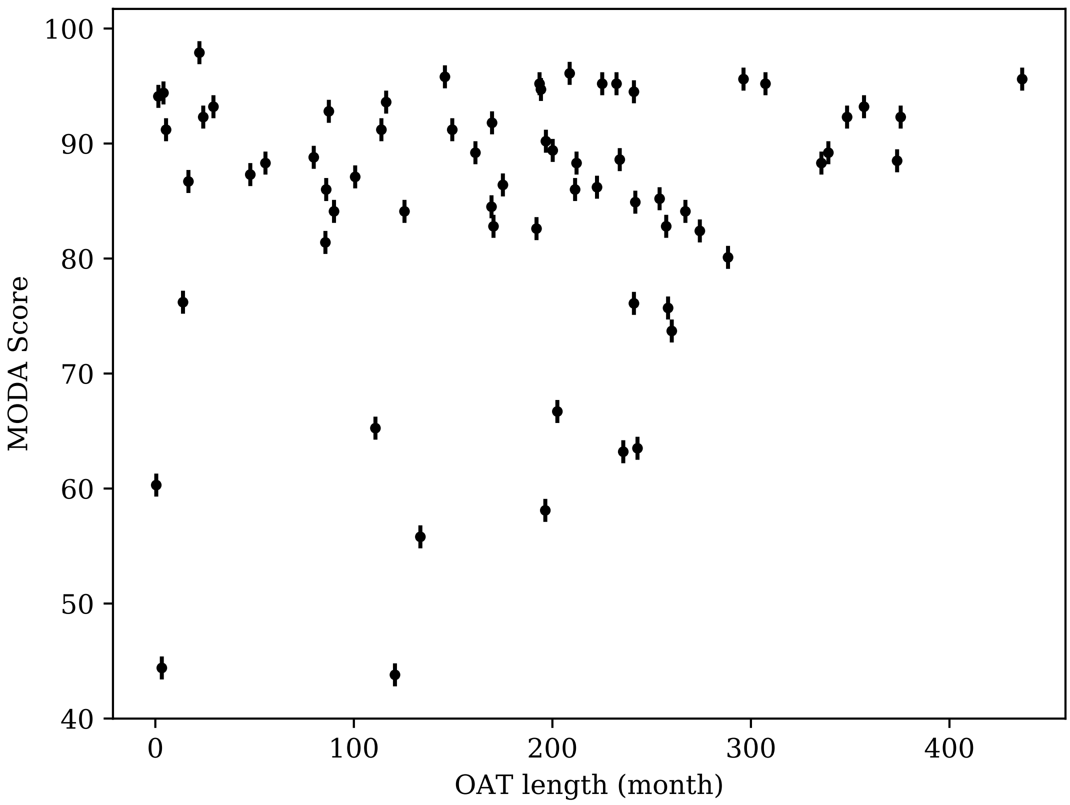

Figure S3. MODA scores do not show a clear dependence on OAT length. For increasing OAT length, MODA scores however tend to be less dispersed and have larger values. This seems to suggest that OAT length does not negatively influence cognitive functions.

3. MODA scores and years of education

When calculating MODA scores, correction tables for the years of school were applied. Unsurprisingly, Fig. S4 does not show any clear relation between MODA scores and years of education. Still, it is remarkable that MODA scores tend to have larger values as the years of education increase. There seems indeed to be a logarithmic-like function with MODA scores dispersion decreasing for increasing years of education. Such a trend is however limited by the MODA scores for 13 years of education being significantly scattered around their mean. A further limitation to this logarithmic-like function is due to the relatively small number of patients with high education in our cohort.

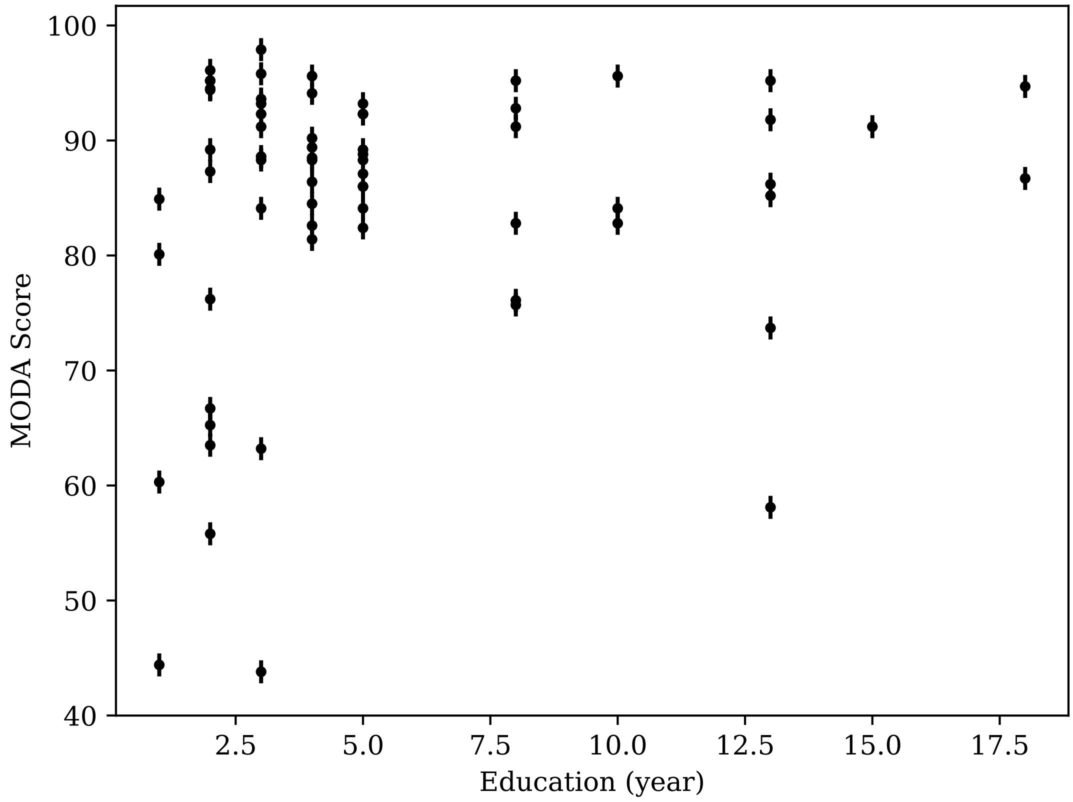

Figure S4. No clear trend can be identified between MODA scores and years of education. However, MODA scores tend to have on average larger values as the years of education increase.

4. MODA scores: mean, standard deviation and standard error per vitamin K centile

Table S1. Mean values, standard deviations and standard errors of MODA scores for vitamin K1 centiles.

Vitamin K1 centile (µg/L)	MODA Mean Value	MODA St. Deviation	MODA St. Error	Patients (number)
0.100-0.199	81	15	4	12
0.200-0.299	84	17	5	11
0.300-0.399	82	12	4	9
0.400-0.499	90	4	1	8
0.500-0.599	90	6	3	4
0.600-0.699	88	0	1	1
0.700-0.799	86	6	4	2
0.800-0.899	89	5	2	4
> 0.900	88	10	4	7

In Table S1, MODA scores have been binned considering vitamin K1 concentration centiles above 0.100 µg/L, as described in the main manuscript. Table S1 shows the mean value, standard deviation and standard error of the MODA scores for each vitamin K1 centile. Further studies on larger cohorts are needed to confirm these findings.

5. MODA scores and vitamin K levels vs comorbidities

Table S2. MODA scores and vitamin K levels according to comorbidities. None: no comorbidity reported apart from the main indication to OAT (see Table 1 in the main manuscript); AD: Alzheimer's disease; CKD: chronic kidney disease; DM: diabetes mellitus; HT: hypertension; NEO: malignant neoplasm; Multiple: more than one of the previous. For both MODA score and vitamin K concentration, the value and the associated error are the mean and the standard error, respectively. For the patient with Alzheimer's disease, the errors on the MODA score and on the vitamin K are given by the uncertainty in the methods as described in the main manuscript.

Comorbidity	MODA Score	Vitamin K (μg/L)	Patients (number)
None	84 ± 2	0.327 ± 0.056	35
AD	13 ± 1	0.048 ± 0.006	1
CKD	87 ± 2	0.456 ± 0.104	23
DM	81 ± 5	0.262 ± 0.094	8
HT	81 ± 8	0.194 ± 0.095	5
NEO	82 ± 6	0.399 ± 0.128	4
Multiple	87 ± 2	0.252 ± 0.056	9

As shown in Table S2, patients have been divided into groups according to their comorbidities. 35 patients did not have any further diseases apart from the main indication to OAT (see Table 1 in the main manuscript). 9 patients had more than one of the concurrent diseases listed in Table S2. Within these 9 patients, the combinations of comorbidities were the following:

Chronic kidney disease and diabetes mellitus (3 patients)

Chronic kidney disease and malignant neoplasm (1 patient)

Hypertension and chronic kidney disease (1 patient)

Hypertension and diabetes mellitus (1 patient)

Hypertension and malignant neoplasm (3 patients)

The mean values for the MODA score and the vitamin K concentration are, within their errors, similar for each group. The only outlier is the MODA score for the patient affected by Alzheimer's disease. Here, the extremely small value for the MODA score is not surprising, but further confirms the validity of the MODA test to identify both mild and severe signs of cognitive deterioration. Interestingly, also the vitamin K concentration is very low in this patient (0.048 ± 0.006 μg/L), thus supporting the hypothesised role of vitamin K in cognitive impairment; the finding is however limited by the LLOQ for the HPLC method being equal to 0.060 μg/L [3].

Figures S5 and S6 graphically show that there is no clear relation between, on the one hand, concurrent diseases and, on the other hand, vitamin K levels and cognitive functions. For any comorbidity, vitamin K levels and MODA scores can potentially take both small and large values. This further supports the relationship between vitamin K levels and MODA scores as neither of them seems to be significantly affected by any major concurrent diseases. A case control study conducted by our group on the relationship between vitamin K and the percentage of time in therapeutic range (TTR%) had also reported that subjects with low vitamin K1 plasmatic concentrations (below 0.060 μg/L) were more likely to show signs of neurodegenerative diseases [4]. Further studies on larger cohorts of patients are however needed in order to definitely confirm the present findings.

As shown in Table S3 and Figs S7-8, the analysis holds true also when excluding patients with vitamin K levels below the threshold value of 0.060 μg/L, corresponding to the LLOQ for the HPLC method [3]. Apart from the data analysis shown in Table S2 and Figs S5-6, all the previous analyses were conducted with such a threshold value. We presented the full data set in Table S2 and Figs S5-6 given the interesting finding of very low vitamin K levels and MODA score for the patient suffering from Alzheimer's disease.

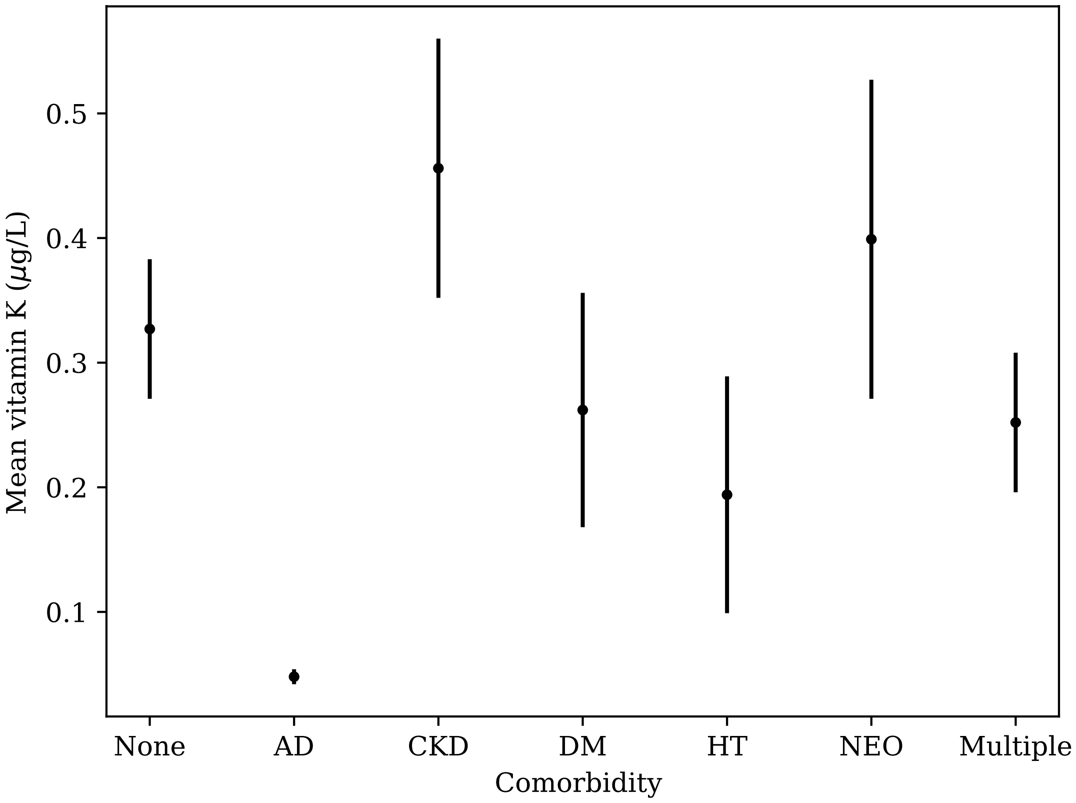

Figure S5. Vitamin K levels do not show any strong dependence on comorbidities. Interestingly, the only outlier is the patient suffering from Alzheimer's disease (AD). In this case, vitamin K levels are significantly lower in comparison with the rest of the cohort. Considering each comorbidity, the value and the associated error for the vitamer concentration are the mean and the standard error, respectively. For the patient with Alzheimer's disease, the error is given by the uncertainty in the method as described in the main manuscript.

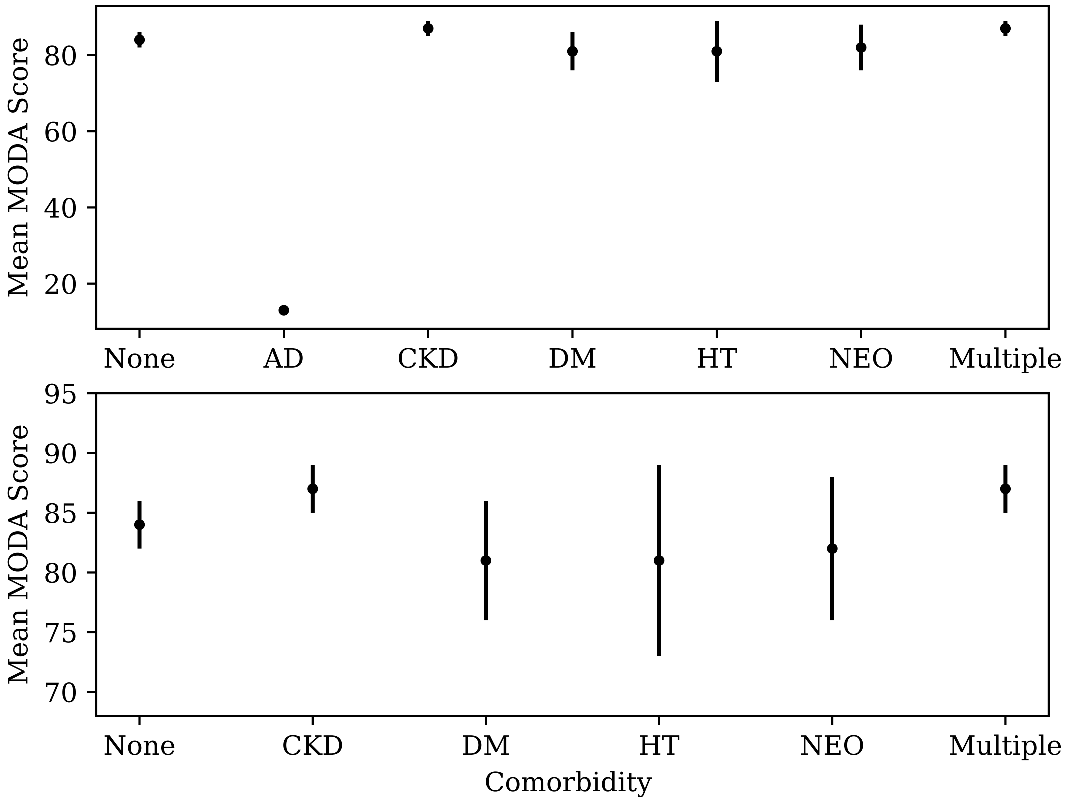

Figure S6. MODA scores do not show any clear dependence on comorbidities. Unsurprisingly, the only outlier is the patient suffering from Alzheimer's disease (AD). In this case, the MODA score, as well as the vitamin K levels (see Table S2 and Fig. S5), is significantly smaller in comparison with the rest of the cohort. The bottom subplot highlights that, excluding the patient with AD, the MODA scores are similar within their errors. Considering each comorbidity, the value and the associated error for the MODA score are the mean and the standard error, respectively. For the only patient with AD, the error on the MODA score is given by the uncertainty in the method as described in the main manuscript.

Table S3. MODA scores and vitamin K levels according to comorbidities. Data has been analysed excluding patients with vitamin K levels below the threshold value of 0.060 μg/L, corresponding to the LLOQ for the HPLC method. The values and the associated errors for the MODA score and the vitamin K concentration are the mean and the standard error, respectively.

Comorbidity	MODA Score	Vitamin K (μg/L)	Patients (number)
None	84 ± 3	0.430 ± 0.064	26
CKD	87 ± 2	0.496 ± 0.110	21
DM	80 ± 6	0.342 ± 0.107	6
HT	75 ± 13	0.314 ± 0.114	3
NEO	82 ± 6	0.399 ± 0.128	4
Multiple	89 ± 2	0.363 ± 0.029	6

Within the 6 patients with more than one comorbidity, the combinations of concurrent diseases were the following:

Chronic kidney disease and diabetes mellitus (3 patients)

Chronic kidney disease and malignant neoplasm (1 patient)

Hypertension and chronic kidney disease (1 patient)

Hypertension and malignant neoplasm (1 patient)

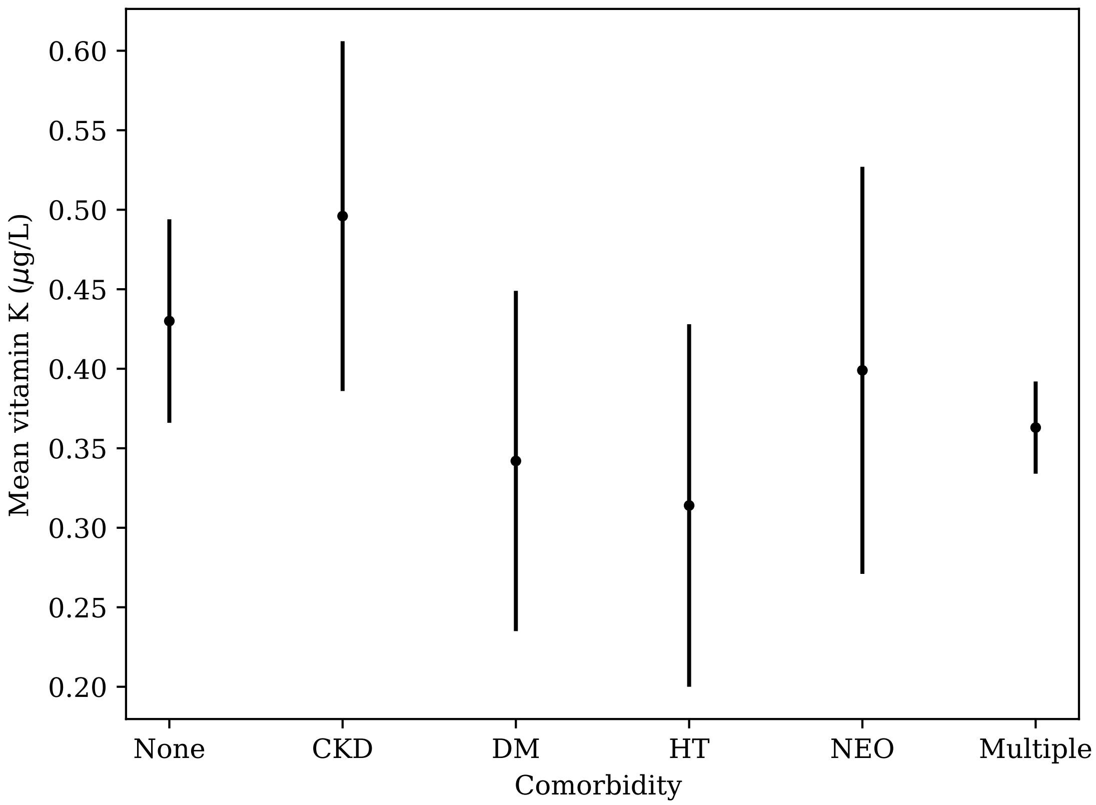

Figure S7. Further analysis on the relation between vitamin K levels and comorbidities, setting a threshold value for the former of 0.060 μg/L. The threshold value corresponds to the LLOQ for the HPLC method, and was applied to all the previous analyses (Figs 1-2 in the main manuscript, and Figs S1-4). Also here, performing the analysis with a threshold value, vitamin levels do not show any clear dependence on concurrent diseases patients may suffer from. The value and the associated error for the vitamin K levels are the mean and the standard error, respectively.

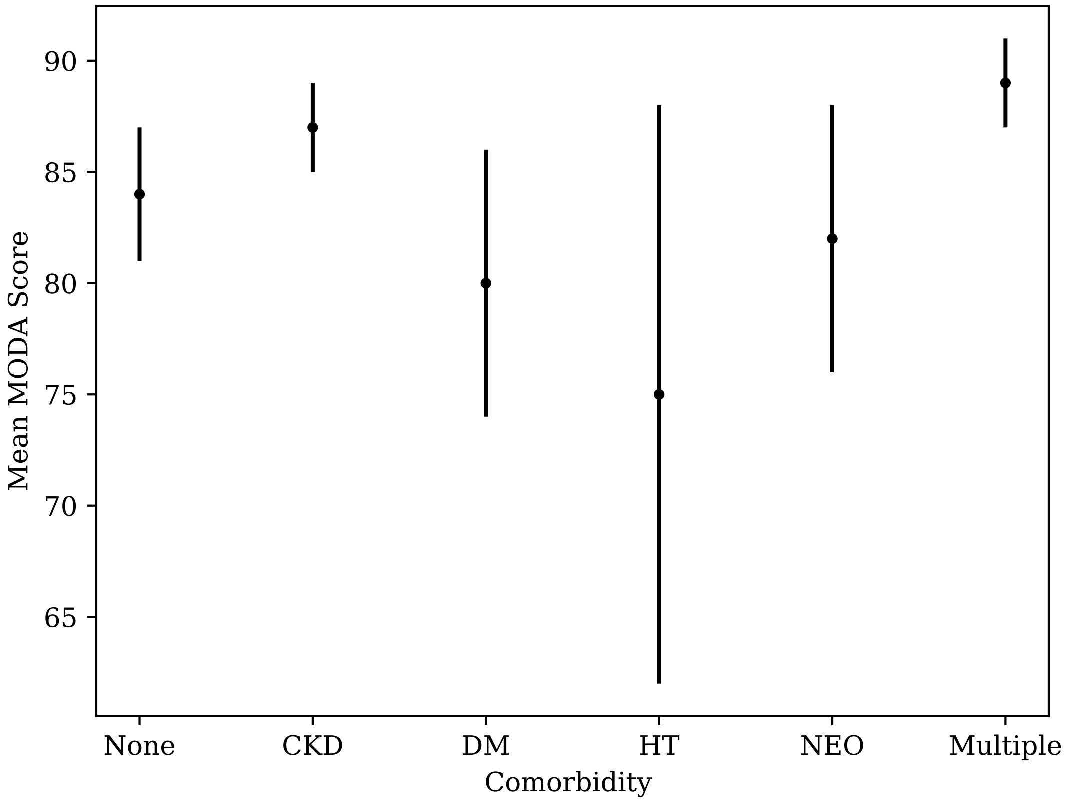

Figure S8. Further analysis on the relation between MODA scores and comorbidities, setting a threshold value for the vitamin K concentration of 0.060 μg/L. The threshold value corresponds to the LLOQ for the HPLC method, and was applied to all the previous analyses (Figs 1-2 in the main manuscript, and Figs S1-4 and S7). Also here, performing the analysis with a threshold value, cognitive functions as measured by MODA scores do not show a clear dependence on comorbidities. The value and the associated error for the MODA score are the mean and the standard error, respectively.

6. Supplementary References

1) Tamadon-Nejad S, Ouliass B, Rochford J, Ferland G. Vitamin K deficiency induced by warfarin is associated with cognitive and behavioural perturbations, and alterations in brain sphingolipids in rats. *Front Aging Neurosci.* 2018; 10: 213.
2) Annweiler C, Denise S, Duval G, Ferland G, Bartha R, Beauchet O. Use of vitamin K antagonists and brain volumetry in older adults: preliminary results from the GAIT study. *J Am Geriatr Soc.* 2015; 63: 2199-202.
3) Gentili A, Cafolla A, Gasperi T, Bellante S, Caretti F, Curini R, Fernández VP. Rapid, high performance method for the determination of vitamin K (1), menaquinone-4 and vitamin K (1) 2,3-epoxide in human serum and plasma using liquid chromatography-hybrid quadrupole linear ion trap mass spectrometry. *J Chromatogr A*. 2014; 1338: 102–10.
4) Cafolla A, Gentili A, Cafolla C, Perez V, Baldacci E, Pasqualetti D, Demasi B, Curini R. Plasma vitamin K1 levels in Italian patients receiving oral anticoagulant therapy for mechanical heart prosthesis: a case-control study. *Am J Cardiovasc Drugs*. 2016; 16: 267-74.
